# Supplementary material for: Pan-Coronavirus Protease Engagement and Differentiated Pharmacology of Antiviral Candidate CMX990
Source: bioRxiv. 2026 Oct 1:2026.09.29.755428. Preprint. [Version 1] doi: 10.64898/2026.09.29.755428 (PMC13648650; doi:10.64898/2026.09.29.755428)
Supplement: Supplementary file 1 [file media-1.pdf]

Supporting Information

for

Pan-Coronavirus Protease Engagement and Differentiated Pharmacology of Antiviral Candidate CMX990

N. G. R. Dayan Elshan[a], Karen C. Wolff[a], Laura Riva[a], Malina A. Bakowski[a], Simeon Babarinde[b], Anshul Chaudhary[b], Mark Zeller[c], Ashley K. Woods[a], Gennadii Grabovyi[a], Katy B. Wilson[a], Rebecca A. Leonard[d], Briana L. McGovern[e], Jared Benjamin[e], Isidora Suazo[e], Mary Gordon[e], Frank O. Weiss[a], Sourav Ghorai[a], James R. Pedroarena[a], Kristi L. Marquardt[c], Maheeka Bimal[c], Stuart Weston[f], Carly Dillen[f], Appaso M. Jadhav[a], Melanie G. Kirkpatrick[a], Amal Gebara-Lamb[a], Nisar Farhat[a], Vân T. B. Nguyen-Tran[a], Victor Chi[a], Kyoung-Jin Lee[a], Case W. McNamara[a], Anil Kumar Gupta[a], Alireza Rahimi[a], John R. Teijaro[c], Jian Jeffrey Chen[a], Sean B. Joseph[a], Matthew Frieman[f], Nicholas S. Heaton[d], Kris White[e], Ian A. Wilson[b], Peter G. Schultz[a][g], Sumit K. Chanda[a], Arnab K. Chatterjee[a]*

*corresponding author: E-mail: dnakath@scripps.edu

[a] Calibr-Skaggs Institute for Innovative Medicines, The Scripps Research Institute, La Jolla, CA, USA

[b] Department of Integrative Structural and Computational Biology, The Scripps Research Institute, La Jolla, CA, USA

[c] Department of Immunology and Microbiology, The Scripps Research Institute, La Jolla, CA, USA

[d] Department of Microbiology and Molecular Genetics, Duke University School of Medicine, Durham, North Carolina, USA

[e] Department of Microbiology, Icahn School of Medicine at Mount Sinai, New York, NY, USA

[f] Department of Microbiology and Immunology, University of Maryland School of Medicine, Baltimore, MD, USA

[g] Department of Chemistry, The Scripps Research Institute, La Jolla, CA, USA

Table of Contents

Figure S1. Sequence alignment of SARS-CoV-1, SARS-CoV-2, and MERS-CoV main proteases **S2**

Figure S2. Electron density maps of CMX990 bound to the active site of coronavirus main proteases ...**S3**

Table S1.Data collection and refinement statistics for CMX990-M^{pro} crystal structures……………..…..**S4**

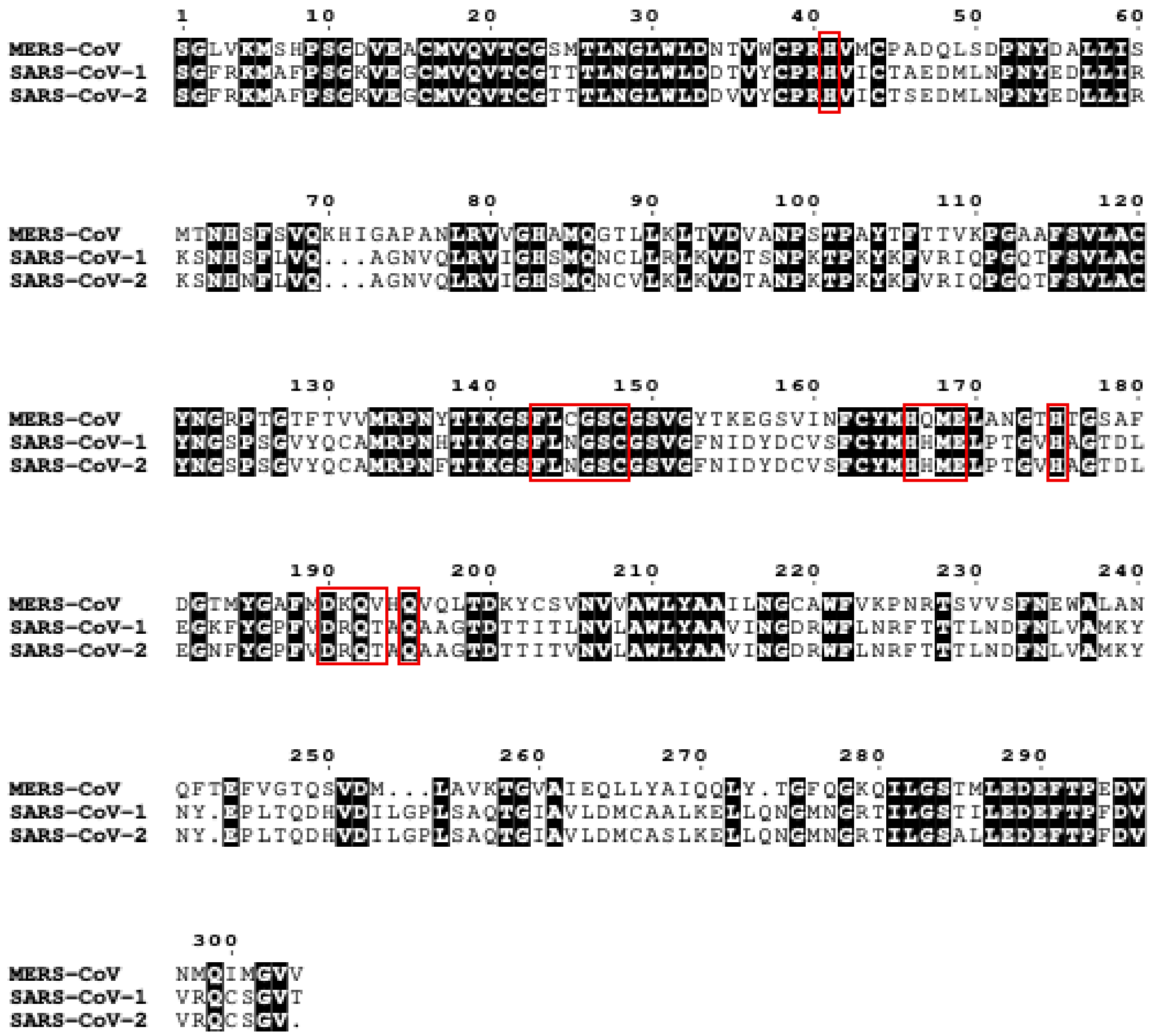

Figure S1. Sequence alignment of SARS-CoV-1, SARS-CoV-2, and MERS-CoV main proteases. Amino acid sequences of main protease (M^{pro}, nsp5) from SARS-CoV-1 (UniProt P0C6U8), SARS-CoV-2 (UniProt P0DTC1), and MERS-CoV (UniProt K9N638) were aligned to assess sequence conservation among the three structurally characterized coronavirus main proteases. Residues located within 4 Å of CMX990 in the SARS-CoV-1 M^{pro}–CMX990 crystal structure are highlighted with red boxes. Conserved residues are shaded according to sequence conservation. Dots indicate gaps introduced by the sequence alignment.

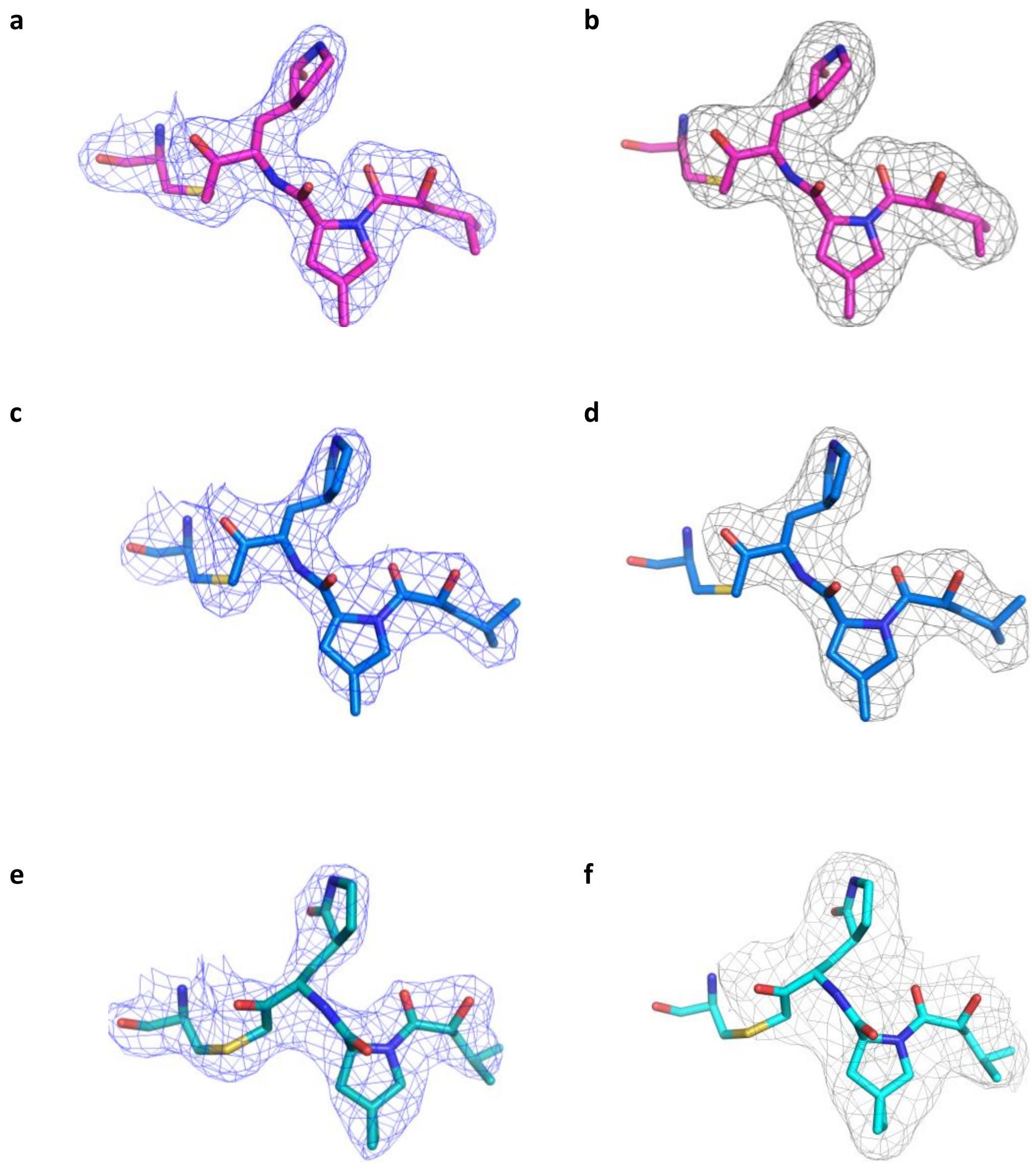

Figure S2. Electron density maps of CMX990 bound to the active site of coronavirus main proteases. (a, c, e) Conventional 2Fo−Fc electron density maps (blue mesh) contoured at 1.0σ for CMX990 bound to SARS-CoV-1, SARS-CoV-2, and MERS-CoV M^{pro}, respectively. (b, d, f) Corresponding polder omit maps (grey mesh), calculated after omission of the ligand (but not the Cys to which the ligand is attached) from the model, contoured at 2.5σ for SARS-CoV-1 and SARS-CoV-2 and 1.0σ for MERS-CoV. CMX990 is shown as sticks.

Table S1. Data collection and refinement statistics for CMX990-M^{pro} crystal structures

**Data Collection**	**SARS-CoV-1 +CMX990**	**MERS-CoV +CMX990**	**SARS-CoV-2 +CMX990**
Beamline	NSLS-II (17-ID-1)	NSLS-II (17-ID-1)	SSRL 12-2
Wavelength (Å)	0.92012	0.92012	0.97946
Space group	P 2_1 2_1 2_1	P 1 $2_1$1	P 2_1 2_1 2_1
Unit cell parameters			
a, b, c (Å)	68.0 68.3 146.9	62.8 112.8 92.1	67.7 101.0 103.4
α, β, ɣ (°)	90 90 90	90 91.5 90	90 90 90
Resolution (Å)[a]	34.1 - 2.20 (2.23 - 2.20)	32.3 - 2.47 (2.50 - 2.47)	50.0 - 2.54 (2.58 - 2.54)
Unique reflections [a]	33,672 (1,726)	46,145 (2,324)	23,492 (931)
Redundancy [a]	7.5 (7.2)	7.2 (7.5)	11.1 (6.0)
Completeness (%) [a]	94.8 (100.0)	99.7 (99.2)	96.9 (77.8)
<I/σI> [a]	7.2 (0.7)	6.9 (1.1)	23.3 (2.8)
R_{sym}[b] (%) [a]	18.3 (>100)	22.9 (>100)	11.0 (51.0)
R_{pim}[b] (%) [a]	7.2 (>100)	9.1 (>100)	3.3 (20.6)
$CC_{1/2}$[c] (%) [a]	99.5 (38.5)	99.5 (34.5)	99.9 (97.2)
**Refinement Statistics**			
Resolution (Å)	34.1 - 2.20	32.3 - 2.47	50.0 - 2.54
Reflections (work)	32,088	43,803	20,824
Reflections (test)	1,572	2,301	1,106
R_{cryst} [d]/R_{free} [e] (%)	20.8 / 25.7	20.7/28.6	20.8 / 25.5
No. of copies in ASU	2	4	2
No. of atoms	4,703	9,541	4,830
M^{pro}	4,522	9,248	4,666
CMX990	58	116	58
Solvent (water)	103	177	94
DTT/GOL[f]	20	-	12 (GOL)
Average B-values of all atoms	58	48	37
M^{pro}	59	47	37
CMX990	55	48	36
Solvent (water)	52	42	33
Wilson B-value (Å^2)	47	37	37
**RMSD for ideal geometry**			
Bond length (Å)	0.008	0.008	0.002
Bond Angle (°)	0.9	1.0	0.5
**Ramachandran statistics (%)**[g]			
Favored	95	94	98
Outliers	1.2	0.17	0.7
**PDB ID**	**38DQ**	**38TR**	**38TV**

[a] Numbers in parentheses refer to the highest resolution shell

[b] R_{sym} = S*hhk* S*i* | I*hkl,i* - <I*hkl*> |/ S*hkl* Si I*hkl*, *i* and R_{pim} = S*hkl* (1/(n-1))1/2 S*i* | I*hkl,i* - <I*hkl*> |/S*hkl,i*, where I*hkl,i* is the scaled intensity of the ith measurement of reflection h, k, l, <I*hkl*> is the average intensity for that reflection, and *n* is the redundancy.

[c] $CC_{1/2}$ = Pearson correlation coefficient between two random half datasets.

[d] R_{cryst} = S*hkl* | *Fo* – *Fc* | /S*hkl* | *Fo* | x 100, where *Fo* and *Fc* are the observed and calculated structure factors, respectively.

[e] R_{free} was calculated as for R_{cryst}, but on a test set comprising 5% or 10% of the data excluded from refinement.

[f] DTT (dithiothreitol) and GOL (glycerol) from purification buffer

[g] From MolProbity.[1]

References: (1) Williams, C. J.; Headd, J. J.; Moriarty, N. W.; Prisant, M. G.; Videau, L. L.; Deis, L. N.; Verma, V.; Keedy, D. A.; Hintze, B. J.; Chen, V. B.; et al. MolProbity: More and better reference data for improved all-atom structure validation. *Protein Sci* **2018**, *27* (1), 293–315. DOI: 10.1002/pro.3330
